# Socioeconomic Inequalities in Disability-free Life Expectancy in Older People from England and the United States: A Cross-national Population-Based Study

**DOI:** 10.1093/gerona/glz266

**Published:** 2020-01-15

**Authors:** Paola Zaninotto, George David Batty, Sari Stenholm, Ichiro Kawachi, Martin Hyde, Marcel Goldberg, Hugo Westerlund, Jussi Vahtera, Jenny Head

**Affiliations:** 1 Department of Epidemiology and Public Health, University College London, UK; 2 Department of Public Health, University of Turku and Turku University Hospital, Finland; 3 Department of Social and Behavioral Sciences, Harvard University, Boston, Massachusetts; 4 Centre for Innovative Ageing, College of Human and Health Sciences, Swansea University, UK; 5 Inserm, Population-based Epidemiologic Cohorts Unit-UMS 011, Villejuif, France; 6 Inserm, Aging and Chronic Diseases, Epidemiological and Public Health Approaches, Villejuif, France; 7 Stress Research Institute, Stockholm University, Sweden

**Keywords:** Disability, Healthy life expectancy, Socioeconomic status, Cross-national

## Abstract

**Background:**

We examined socioeconomic inequalities in disability-free life expectancy in older men and women from England and the United States and explored whether people in England can expect to live longer and healthier lives than those in the United States.

**Methods:**

We used harmonized data from the Gateway to Global Aging Data on 14,803 individuals aged 50+ from the U.S. Health and Retirement Study (HRS) and 10,754 from the English Longitudinal Study of Ageing (ELSA). Disability was measured in terms of impaired activities and instrumental activities of daily living. We used discrete-time multistate life table models to estimate total life expectancy and life expectancy free of disability.

**Results:**

Socioeconomic inequalities in disability-free life expectancy were of a similar magnitude (in absolute terms) in England and the United States. The socioeconomic disadvantage in disability-free life expectancy was largest for wealth, in both countries: people in the poorest group could expect to live seven to nine fewer years without disability than those in the richest group at the age of 50.

**Conclusions:**

Inequalities in healthy life expectancy exist in both countries and are of similar magnitude. In both countries, efforts in reducing health inequalities should target people from disadvantaged socioeconomic groups.

Life expectancy has increased dramatically in the United States and United Kingdom over the last century, however, new evidence emerged showing that in more recent years life expectancy at birth has been decreasing in the United States (1) and leveling off in the United Kingdom (2). Simultaneously, increases in the prevalence of disability and chronic conditions among older adults have been reported in the United States (3), England (4), and globally (5), bringing financial challenges for governments and health care systems worldwide.

While life expectancy is a useful indicator of health, it is becoming increasingly recognized that quantification of the quality of remaining years of life is also crucial (6). Health expectancy gives an estimate of the number of years of life spent in favorable states of health or without disability. Health expectancy measures have value for monitoring time trends and inequalities in population health because they combine data on both mortality and morbidity or disability (7). While health differences between older Americans and older English people are well documented (8–11), direct comparisons between these two countries in healthy life expectancy at older ages are less documented.

It is now well established that in the United States and England, there are striking socioeconomic inequalities, in both general health and life expectancy (8,9,12–18), with apparent socioeconomic gradations, rather than differences only being seen between rich and poor. While these studies show differences between countries in health and socioeconomic differences within each country in health expectancy, differences between these two countries in socioeconomic inequalities in health expectancy have not yet been investigated. Health care coverage in England is publicly funded, whereas in the United States, it is provided through private health insurance (for many working-age adults) and through publicly funded health insurance for the poor (Medicaid) as well as those aged 65 years and older. With the introduction of the Affordable Care Act, the number of uninsured individuals in the United States declined (19), however, economically disadvantaged individuals and those in worse health still have more difficulty accessing health care. Lack of health insurance among those aged below 65 years does not only affect prevention and early diagnosis, but also access to appropriate medical intervention when serious illness strikes and the ability to pay for other needs, such as adequate housing and food. Furthermore, since access to health care is not the only explanation for inequalities in health (20), cross-national comparisons of health expectancy can also help evaluating strategies adopted in different countries to help reducing health inequalities. Other possible explanations for greater health inequalities in the United States compared to England (10) might relate to a more generous welfare state system in England compared to the United States, including unemployment compensation, sick pay, housing policies, and social retirement benefits. These contextual factors can in turn provide better psychosocial health and reduced stress, especially among socioeconomically disadvantaged groups. It is possible that older Americans are exposed to more psychosocial distress, which has been shown to increase the risk of chronic conditions and early mortality (21).

The health indicators that have commonly been used to compute health expectancy include, among others, self-rated health (referred to as “healthy life expectancy”), activities of daily living (ADL) and/or instrumental activities of daily living (IADL) (referred to as “disability-free life expectancy”), and chronic morbidity (referred to as “chronic disease-free or morbidity-free life expectancy”) (17,22–24). However, self-rated health is based on subjective health status and identical questions may not mean the same to people across different cultures. Disability-free life expectancy is one of the most widely accepted measure of health expectancy (22) and has been recommended in cross-country comparative studies (17) because it is less sensitive to cultural factors.

Lack of harmonization of both health and socioeconomic measures is often one of the main obstacles for comparing health expectancies between countries (17,22). However, the Health and Retirement Study (HRS) in the United States (25) and the English Longitudinal Study of Ageing (ELSA) (26,27) were expressly designed to be comparable, hence providing a unique opportunity for cross-national comparisons of inequalities in health expectancy. In exploring inequalities in health in later life, the use of a range of socioeconomic indicators has been recommended (28), however, most studies of inequalities in health expectancy have focused on education and occupational social class (22). Given the link between lifetime socioeconomic status and health (29,30), the use of several measures of socioeconomic position that can capture different stages of life may yield a better understanding of how socioeconomic status relates to late life health and longevity. In particular, wealth is considered a useful indicator of the long-term socioeconomic status of older people since it captures both past and present circumstances (28,31,32).

Although it is well established that individuals in lower socioeconomic groups in England have better health that people in higher socioeconomic groups in the United States (8,10), the extent to which these socioeconomic differences between the two countries extend to healthy life expectancy is not known. The aim of this study was to explore whether the health disadvantage of older Americans extends to healthy life expectancy. Furthermore, we explored the extent to which inequalities in healthy life expectancy within each country differed according to different measures of socioeconomic status. We used socioeconomic indicators selected from different stages of life namely, education, wealth, and social class.

## Methods

### Data

We used data from two prospective cohort studies of ageing: ELSA and HRS. Established 10 years after HRS, ELSA was designed to be comparable in terms of population sampling, periodicity, and content (including the specific wording of questions) (33,34). The two studies have been described in detail elsewhere (25,26). Briefly, HRS (25) is a nationally representative biennial longitudinal survey of people aged 51 years and older that began in 1992 and currently includes more than 37,000 individuals. Since 1992 HRS has grown to represent all Americans aged 50 years and older (25). The sample selection was based on a multistage area probability design involving geographical stratification and clustering. Baseline data collection is through face-to-face interviews and follow-up interviews rotate between face-to-face and telephone administration (for those aged less than 80 years). National representation of this population is maintained over time using a steady state design which adds a new cohort of persons entering their 50’s every 6 years.

ELSA (26,27), is also an open-access, nationally representative, biennial longitudinal survey of those aged 50 years and older living in private households in England that began in 2002/2003. The sample was drawn from participants in the Health Survey for England (HSE), an annual cross-sectional survey that is designed to monitor the health of the general population (35). For the first wave (2002/2003), participants were recruited from the HSE by using a two-stage stratified random sampling process. Data were also collected through face-to-face interviews at each wave. Comparisons of the sociodemographic characteristics of participants against results from the 2011 national census indicate that the sample was broadly representative of the English population (26). Informed consent was obtained from all individual participants included in the study.

To maximize comparability, we used harmonized data files, from 2002/2003 to 2012/2013, available from *The Gateway to Global Aging Data* (g2aging.org) which is a data and information platform developed to facilitate cross-country analyses. The data files provided a set of harmonized or identically defined variables.

In both cohorts, we included people aged 50 years or older with valid data on health and socioeconomic status, resulting in analytical samples of 10,754 (out of the 11,391 ELSA members in 2002/2003) and 14,803 (out of the 17,758 HRS members in 2002 aged 50 years and older) (refreshment samples added after 2002 are excluded from these analyses).

### Socioeconomic Indicators

In both countries, total household wealth (10) (sum of net financial wealth and net housing wealth less all debts) was divided into three groups (ie, each containing 33% of the sample). Educational attainment (10) was defined as: low (less than high school), medium (high school graduate and some college), and high (college or more). Based on the Standard Occupational Classification 2000, we defined occupational social class (36) as high (managers, professionals, associate professionals, or technical occupations), intermediate (administrative and secretarial, personal services, sales), and low (routine, manual, elementary occupations), with those who never worked or long-term unemployed being excluded. All variables were measured in 2002 (for some people, social class was measured before 2002; therefore, information from earlier waves was used).

### Outcome Measures

We measured health expectancy using the presence of disability. At each wave in both studies, all participants were asked whether they had difficulties in performing activities of daily living (ADL) (eg, dressing, walk across a room, bathing or showering, eating, getting in/out of bed, using the toilet) and instrumental activities of daily living (IADL) (eg, using a map, preparing a hot meal, shopping for groceries, making phone calls, taking medications, managing money). Responses were summed and categorized as no disability (0 or 1 ADL/IADL) and disability (2+ ADL/IADL). The cutoff of 2 or more ADL or IADL was chosen based on the average number of ADL or IADL limitations reported by people (in ELSA) who at baseline were in receipt of health or disability benefits. Health expectancy based on disability is named here as disability-free life expectancy.

Mortality up to March 2013 was ascertained from linked register data for ELSA and through linkages to the National Death Index and reports from survivors for HRS.

### Statistical Analyses

Total length of time in study was 10 years (from 2002/2003 to 2012/2013, average follow-up 6 years); by the end of follow-up period, 4,275 deaths occurred in the U.S. sample and 2,470 in the English sample.

We used discrete-multistate life table models (17,37,38) suitable for longitudinal data to estimate health expectancy for the ages of 50–100. We defined the following three health states: healthy, unhealthy, and dead. There were four possible transitions between the health states, namely: healthy to unhealthy (onset), unhealthy to healthy (recovery), healthy to dead, unhealthy to dead.

To estimate multistate life table functions, we used the Stochastic Population Analysis for Complex Events (SPACE) program (37) in SAS 9.2. There are two main components to this program: the data component, which prepares the input datasets and the statistical component, in which transition probabilities and the multistate life table functions and their variances are estimated. Specifically, during the data component, age-specific transition probabilities for all possible transitions are estimated from the data using multinomial logistic regression conditional on age and sex (included as covariates in the model). Health expectancies for the age of 50–100 are then calculated based on these estimated transition probabilities using a stochastic (micro-simulation) approach. By using micro-simulation, it is possible to simulate the life paths of the members of the population in order to derive several summary statistics of the population dynamics. For each study separately, the program generated individual trajectories for a simulated cohort of 100,000 persons with distributions of covariates at the starting point based on the observed study-specific prevalence by 5-year age group, sex, and socioeconomic indicators. Variability measures for these multistate life table estimates (variances, standard errors, and corresponding 95% confidence intervals) are computed using a bootstrap method with 500 replicates for the whole analysis process (multinomial analysis and simulation steps). Analyses were weighted by nonresponse weights.

### Sensitivity Analyses

To assess the influence of race on the results, we computed estimates of healthy life expectancy by socioeconomic indicators in white people only for both countries.

To further assess whether our results were driven by the different distribution of the samples for social class and education, we computed the relative index of inequalities scores for these socioeconomic indicators. The scores were defined as the cumulative rank of each social class or education group (39), based on proportions in each group (which is equal to the proportion of participants in higher social class groups plus one half of the proportion of participants within their own social class group). This takes into account of the relative size of groups in the two countries. The relative index of inequalities score was entered as a linear term in the model, and then the healthy life expectancy was estimated for relative index of inequalities scores of 0.85 (low), 0.5 (median), and 0.15 (high).

## Results

Baseline characteristics for men and women in each country are presented in Table 1. Men and women in England were slightly younger and more than 97% were of white origins compared to 88% in the United States. In the United States, the majority of the sample reported middle social class and medium education, whereas in England, the majority of men and women reported being in a low occupational grade and low education category. The prevalence of disability was 13% in English men and 16% in women slightly higher than in the United States (10% men and 13% women, *p*-value for the country difference in each sex <.001).

**Table 1.** Baseline Characteristics of Participants in ELSA and HRS, England and the United States (2002–2003)

	Men		Women	
	England (ELSA)	United States (HRS)	England (ELSA)	United States (HRS)
**Sample size**	4,980	6,805	5,774	7,998
**Mean age (*SD*)**	64.3 (10.5)	66.6 (11.0)	65.7 (11.7)	66.5 (11.4)
**Ethnicity**	%	%	%	%
White	96.7	88.2	98.1	86.3
**Social class**				
Low grade	52.4	28.2	31.1	11.7
Middle grade	10.3	37.0	46.5	58.0
High grade	37.3	34.8	22.4	30.3
**Wealth**				
Poorest	34.8	25.5	38.4	31.6
Middle	33.0	34.2	33.1	34.5
Richest	32.2	40.3	28.5	33.9
**Education**				
Low	48.9	26.6	56.0	22.8
Medium	36.0	47.1	36.2	59.0
High	15.1	26.3	7.8	18.2
**Disability** ^**a**^				
No	87.4	90.0	84.2	86.6
Yes	12.6	10.0	15.8	13.4

*Note*: ELSA = English Longitudinal Study of Ageing; HRS = Health and Retirement Study; SD = Standard deviation.

^a^Two or more limitations with instrumental/activities of daily living.

Total life expectancy and disability-free life expectancy estimates by gender at the age of 50, 60, 70, and 80 in the United States and England are reported in Table 2. Estimates of total life expectancy and disability-free life expectancy (not adjusted) at all ages were very similar in women in both countries (life expectancy in women was 34.8 years in England and 34.7 in the United States and disability-free life expectancy 28.5 years in England and 28.6 in the United States). For men, estimates of total life expectancy at the age of 50 and 60 were very similar in the two countries, for example, men aged 50 years in England could expect to live an additional 31.3 years and men in the United States 31.5, of these, 26.9 and 27.2 years, respectively, will be spent without disability. At the age of 70 and 80 years, older American men could expect to live up to 1 year longer than older English men. Estimates of average number of years expected to live without disability, at the age of 60, 70, and 80, were slightly higher in men in the United States than men in England. Women in England and the United States could expect to live longer than men, however, disability-free life expectancy at the age of 80 was the same in men and women in both countries.

**Table 2.** Total Life Expectancy and Disability-free Life Expectancy by Age and Sex, England and the United States (2002–2013)

Total life expectancy	Men		Women	
	England (ELSA) Years (95% CI)	United States (HRS) Years (95% CI)	England (ELSA) Years (95% CI)	United States (HRS) Years (95% CI)
Age 50	31.3 (30.7; 31.8)	31.5 (31.0; 31.9)	34.8 (34.2; 35.4)	34.7 (34.2; 35.2)
Age 60	22.2 (21.6; 22.6)	22.7 (22.3; 23.1)	25.5 (24.9; 26.0)	25.5 (25.0; 25.9)
Age 70	14.2 (13.8; 14.6)	15.0 (14.7; 15.3)	16.9 (16.5; 17.4)	17.4 (17.0; 17.7)
Age 80	8.1 (7.9; 8.5)	9.1 (8.8; 9.4)	10.3 (10.0; 10.6)	10.7 (10.4; 11.0)
**Disability-free life expectancy**				
Age 50	26.9 (26.4; 27.4)	27.2 (26.7; 27.7)	28.5 (27.9; 29.0)	28.6 (28.0; 28.9)
Age 60	18.1 (17.6; 18.5)	18.8 (18.5; 19.1)	19.6 (19.1; 20.1)	19.5 (19.1; 19.9)
Age 70	10.8 (10.4; 11.2)	11.5 (11.2; 11.8)	11.6 (11.3; 12.1)	12.0 (11.7; 12.4)
Age 80	5.5 (5.2; 5.9)	6.2 (5.9; 6.4)	6.2 (5.9; 6.5)	6.2 (5.9; 6.6)

*Note*: CI = Confidence interval; ELSA = English Longitudinal Study of Ageing; HRS = Health and Retirement Study.

**Figure 1.**
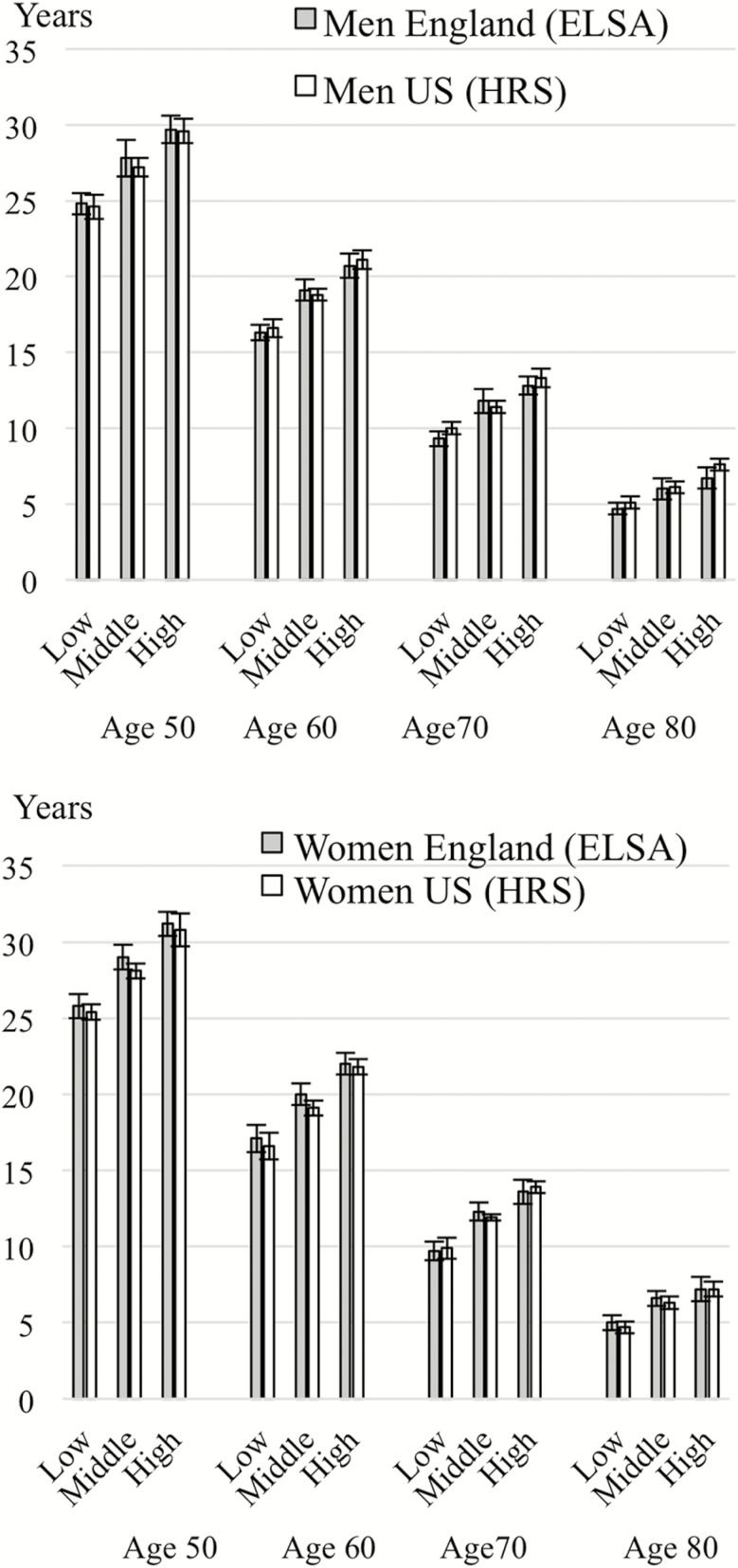
Disability-free life expectancy estimates according to social class and age, men and women in England and the United States (2002–2013).

In Figure 1, we show estimates of disability-free life expectancy for men and women at the age of 50, 60, 70, and 80 according to social class. Socioeconomic differentials in disability-free life expectancy were apparent in both countries at each age and showed similar levels of magnitude: at the age of 50 men and women in the lowest social class group compared to those in the highest group could expect to live 5 fewer years (absolute differences) free from disability in England and the United States. In both countries and both genders, the absolute difference in disability-free life expectancy between the lowest and highest social class decreased with age. Within each socioeconomic group and at all ages, disability-free life expectancy estimates were very similar in the two countries.

**Figure 2.**
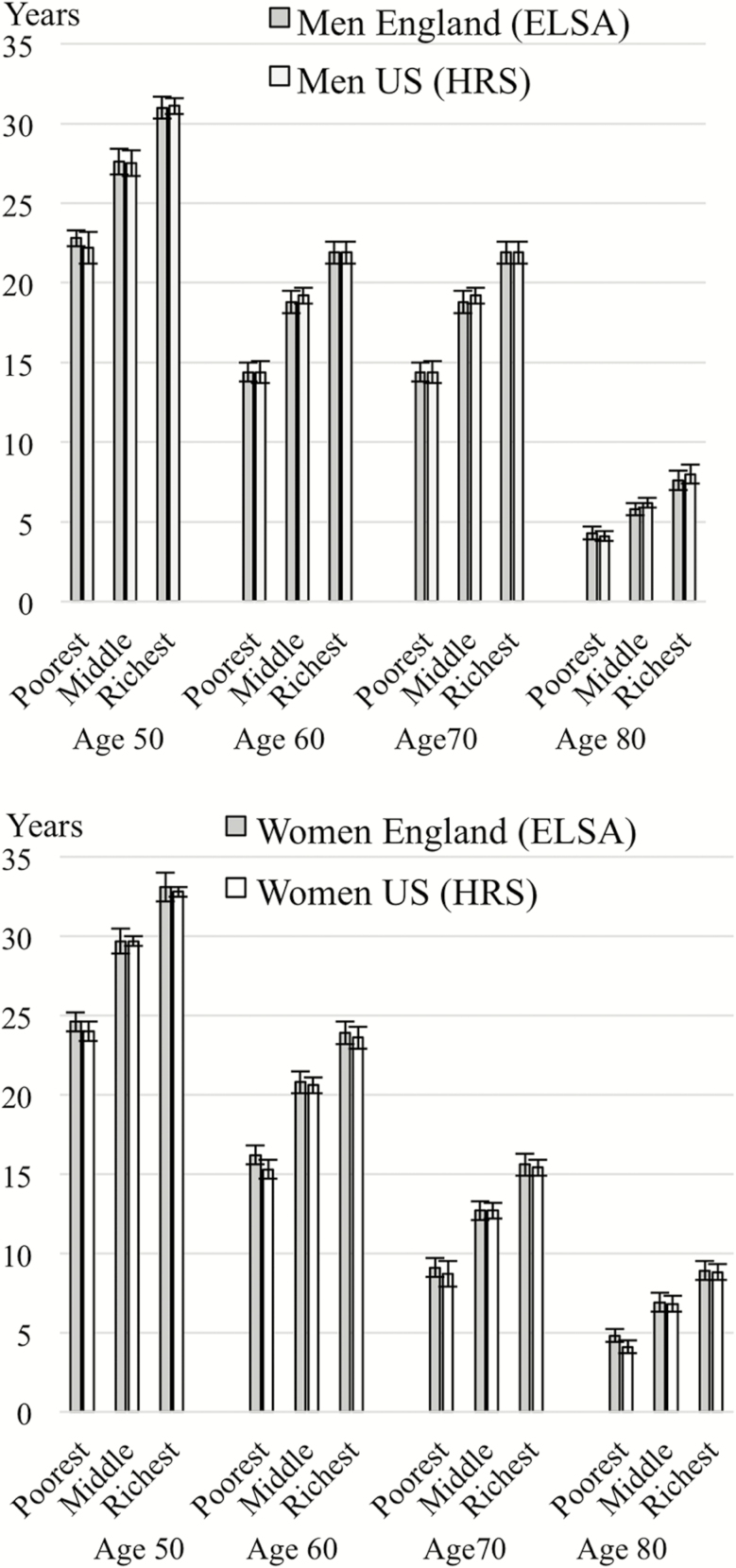
Disability-free life expectancy estimates according to wealth and age, men and women in England and the United States (2002–2013).

In Figure 2, we show estimates of disability-free life expectancy for men and women at the age of 50, 60, 70, and 80 according to wealth. In both countries, at the age of 50, men and women in the richest wealth groups could expect to live an additional 8–9 years free from disability compared to those in the poorest groups (31.0 and 31.1 years for wealthiest men in England and the United States, respectively, vs 22.8 and 22.2 years in men in England and the United States in poorest wealth groups; 33.1 and 32.8 years for wealthiest women in England the United States, respectively, vs 24.6 and 24.0 years in women in England and the United States in poorest wealth groups).

Estimated years expected to live without disability decreased at the age of 60, 70, and 80 in each wealth group, but the socioeconomic gradient remained and the absolute difference between richest and poorest was 7–8 years at the age of 60, 6–7 years at the age of 70 and 3–5 years at the age of 80. Within each wealth group and at all ages, disability-free life expectancy estimates were very similar in the two countries.

**Figure 3.**
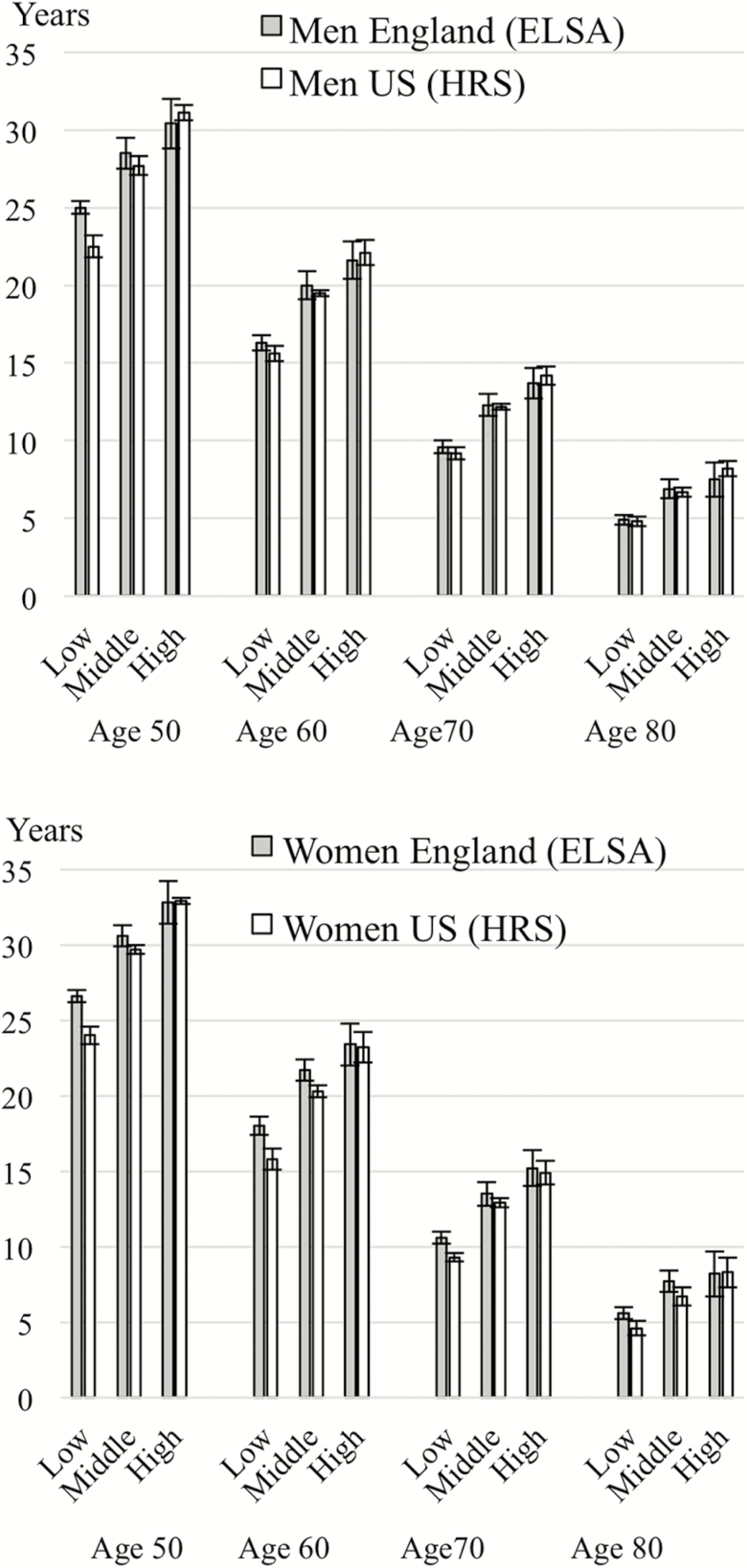
Disability-free life expectancy estimates according to education and age, men and women in England and the United States (2002–2013).

The gradient in additional years of disability-free life expectancy is less marked by education in England compared to the United States (Figure 3). At the age of 50, we observed an absolute difference of 5–6 years between those with low education and those with high education in England, whereas the difference was 9 years in the United States. At older ages, the difference between the most and least educated reduced, but remained significant. Within each education group and at all ages, disability-free life expectancy estimates were very similar in the two countries.

Of the socioeconomic indicators, the absolute difference in disability-free life expectancy was largest for wealth in England and wealth and education in the United States, such that people in the most advantaged groups could expect to live longer without disability than those in the most disadvantaged groups.

Results of the sensitivity analyses are presented in the Supplementary Material. Estimates of disability-free life expectancy for white people only by socioeconomic indicators (Supplementary Tables 1–3) were very similar to those obtained for the sample that included non-white ethnicities (Figures 1–3). A few exceptions should be mentioned: the estimates by social class restricted to white people were slightly higher in low social class groups for men and women in the United States and the absolute difference between higher and lower social class was slightly smaller than that found in the whole sample. The results by education among white people also showed some small differences: men and women in the United States at the age of 50 and 60 in low education groups had slightly higher disability-free estimates compared to results in Figure 3. Furthermore, the absolute difference in disability-free life expectancy between the lowest and the highest education groups was 1 year higher for men and women in England at the age of 50 and 60 and 1 year lower for men and women in the United States at the age of 50 (and 80 for women only).

In Supplementary Tables 4 and 5 in the Supplementary Material, we report estimates of disability-free life expectancy according to the relative index of inequalities scores for social class and education. With a few exceptions, estimates are similar to those reported in Figures 1 and 3, suggesting that overall the distribution of socioeconomic indicators within each country did not affect the results. A few differences should be reported: the absolute difference between the highest and lowest social class groups in England was 1 year larger for men at the age of 50 and 60 and, for women at the age of 50 than the absolute difference reported in Figure 1, whereas in the United States, for men, the absolute difference was smaller by 1 year at the age of 60 and 80. For each education group, the difference between the United States and England in estimates of disability-free life expectancy at the age of 50 and 60 reduced when RII was employed. Furthermore, the absolute difference between the highest and lowest education groups in England was 1 year larger for men at the age of 50 and 60 and, for women at the age of 50, whereas in the United States the absolute difference was 1 year smaller at the age of 50 and 60 for men and at the age of 50 and 80 for women.

## Discussion

Using two large nationally representative samples of older individuals from the United States and England followed from 2002 until 2013 we showed, for the first time, that estimates of total life expectancy and disability-free life expectancy among women in the United States and in England (from HRS and ELSA) are nearly the same, at the age of 50, 60, 70, and 80, respectively. Among men, we observed similar estimates of total life expectancy at the age of 50 and 60 and at the age of 50 for disability-free life expectancy. There was a small advantage of up to 1 year for older American men at the age of 70 and 80 compared to older English men. Similarly, older men in the United States could expect to live approximately half a year longer without disability than men in England at the age of 60, 70, and 80. However, this apparent advantaged was no longer present in the estimates of disability-free life expectancy by socioeconomic status.

Furthermore, we showed that within each country, there was a consistent advantage for people in high socioeconomic groups, particularly for wealth and education, so that they could expect to live a higher number of years without disability. We found that within each country, inequalities in disability-free life expectancy were of similar magnitude. The absolute differences in disability-free life expectancy by socioeconomic indicators decreased with age. A possible explanation is mortality selection, which indicates that when mortality at younger ages is high, it affects frail people first; therefore, survivors at older ages are a selected group of healthier people (40). Another possible explanation is the “age-as-leveler” hypothesis indicating that health inequalities decrease at older ages (41).

Among the socioeconomic indicators considered, wealth generated the greatest disparities in disability-free life expectancy in England and the United States.

Given the novelty of our study, direct comparisons with previous studies is not possible. Nevertheless, our observation that wealth revealed greatest disparities in disability-free life expectancy is in line with previous studies exploring socioeconomic inequalities in health (32,42). Wealth is a measure of lifetime economic advantage or disadvantage, reflecting both past and current circumstances because it comprises of assets from inheritance, lifelong income, as well as patterns in spending and saving. Higher wealth gives access to better housing, healthier life styles as well as better health services.

Our results of education and social class inequalities in disability-free life expectancy within England and the United States are in accordance with those found in previous studies (12–15).

To the best of our knowledge, this is the first study to provide direct comparisons between older adults in England and the United States of socioeconomic inequalities in disability-free life expectancy. A typical problem of comparisons of health expectancies between countries is the lack of harmonization of health measures. Therefore, a major strength of our study is that it draws upon studies specifically designed to be comparable, hence providing high-quality harmonized data of multiple measurements of disability over time. Our method of estimating disability-free life expectancy used discrete multistate life table models applied to longitudinal data. The multistate life tables method has several advantages: it is based on incidence measures representing current health transitions; it allows movement in both directions between all surviving health states; and it allows death rates to differ by health state therefore it takes into account the different mortality profiles by health status. In addition, we used a broad selection of indicators of socioeconomic status from different stages of life. Thus, our study makes a unique contribution in understanding the levels of inequalities in health expectancies between England and the United States. Our results can be generalized to the English and American population of older individuals.

A possible limitation of our study might be related to the fact that participants in longitudinal studies tend to be healthier than those in the general population, thus mortality rates may not match those observed nationally in the United States and England. If that is the case, it is possible that we have overestimated life expectancy and disability-free life expectancy. To further explore this possibility, we compared our estimates of total life expectancy with national life tables (43,44), and found similar results for England and slightly higher estimates for the United States compared to life tables. Although we used harmonized data, some differences between the countries in the distribution of education and social class were found. To further assess whether our results were driven by the different distribution of the samples for social class and education, we computed the relative index of inequalities scores for these socioeconomic indicators. We found that for education the difference in disability-free life expectancy estimates between the two countries reduced slightly; the absolute difference between the top and lowest education groups increased up to 1 year for England and decreased for the United States. Lastly, although both studies have been shown to be nationally representative (25,26,45), they might not totally reflect the diversity of their respective populations. In HRS, oversamples of Asians Americans have not been included; however, according to the U.S. Census 2000, only 2.6% percent of all persons aged 65 years and over were Asians. In ELSA, the prevalence of non-white people is very small, however, comparisons of the sociodemographic characteristics of participants against results from the 2011 national census indicate that the sample was broadly representative of the English population (26). Furthermore, estimates of disability-free life expectancy by socioeconomic indicators among white people only were, as expected, slightly higher in both England and the United States and inequalities were up to 1 year larger in England and up to 1 year smaller in the United States. Overall, the conclusions remained the same.

## Conclusions

Levels of disability-free life expectancy in the United States and in England (from HRS and ELSA) are approximately the same. We have shown that in England and the United States, despite living longer lives, not all the increased years of life are being spent in optimal health. Our findings have implications for policy makers interested in reducing health expectancy inequalities. Improving both the quality and the quantity of years that individuals are expected to live has implications for public expenditure on health, income, and long-term care need of older people as well as work participation in older ages. Furthermore, our results of similar levels of socioeconomic disadvantage in health expectancy in England and the United States suggest that in both countries greater efforts should be put into reducing health inequalities. As suggested by the Marmot review (18), such efforts should be placed into improving opportunities across the social determinants of health: education, occupation, income, home, and community.

## Ethical Approval

Ethical approval for HRS was obtained by the relevant committees at the University of Michigan and the U.S. National Institute on Aging, the primary sponsor of HRS. For ELSA, ethical approval and experimental protocols were granted by the Multi-centre Research and Ethics Committee (MREC). Respondents in HRS and ELSA gave their informed consent to participate in the study and for data linkage.

## Funding

This work was supported by the Academy of Finland (projects 286294 and 294154 for SS). The English Longitudinal Study of Ageing is supported by the National Institute on Aging (grant numbers: 2RO1AG7644 and 2RO1AG017644-01A1) and a consortium of the UK government departments coordinated by the National Institute for Health Research. The funding bodies had no role in the study design; in the collection, analysis, and interpretation of data; in the writing of the manuscript; and in the decision to submit the manuscript for publication.

## Author Contributions

All authors designed and conducted the study. P.Z. analyzed the data with support from J.H.. All authors wrote the manuscript.

## Conflict of Interest

None reported.

## Supplementary Material

glz266_suppl_Supplementary-Material
