# Supplementary material for: Socioeconomic Inequalities in Disability-free Life Expectancy in Older People from England and the United States: A Cross-national Population-Based Study
Source: J Gerontol A Biol Sci Med Sci. 2020 Jan 15;75(5):906–13. doi: 10.1093/gerona/glz266 (PMC7164527; doi:10.1093/gerona/glz266)
Supplement: glz266_suppl_Supplementary-Material [file glz266_suppl_supplementary-material.pdf]

Supplement

eTable 1 Disability-free life expectancy estimates according to social class in white people, England and United States 2002-2013

	Men		Women	
	**England (ELSA) Years (95%CI)**	**US (HRS) Years (95%CI)**	**England (ELSA) Years (95%CI)**	**US (HRS) Years (95%CI)**
**Age 50**				
Low grade	24.7 (23.8; 25.0)	25.4 (24.2; 26.6)	25.8 (25.2; 26.4)	26.1 (24.8; 27.4)
Middle grade	28.0 (26.8; 28.3)	27.6 (26.8; 28.4)	28.5 (27.0; 30.0)	29.1 (28.5; 29.6)
High grade	29.6 (29.2; 30.3)	29.0 (29.1; 29.8)	29.3 (28.6; 30.4)	31.1 (30.9; 32.3)
**Age 60**				
Low grade	16.2 (15.7; 16.5)	17.4 (16.4; 17.8)	17.3 (16.9; 17.7)	17.4 (16.6; 18.3)
Middle grade	18.9 (17.7; 19.7)	19.3 (18.6; 19.8)	20.1 (19.6; 20.7)	20.1 (19.7; 20.7)
High grade	20.6 (20.3; 21.2)	21.2 (20.6; 21.8)	21.9 (21.5; 23.2)	22.3 (21.3; 22.6)
**Age 70**				
Low grade	9.4 (8.9; 9.7)	10.4 (9.9; 10.7)	9.8 (9.4; 10.5)	10.3 (9.5; 11.2)
Middle grade	11.9 (11.3; 12.3)	11.9 (11.3; 12.5)	12.4 (11.8; 13.0)	12.4 (12.0; 13.0)
High grade	12.7 (12.3; 13.1)	13.3 (12.7; 13.8)	13.4 (13.2; 14.4)	14.0 (13.3; 14.4)
**Age 80**				
Low grade	4.8 (4.1; 4.9)	5.3 (5.0; 5.7)	5.1 (4.8; 5.3)	5.1 (4.5; 5.8)
Middle grade	6.0 (5.4; 6.6)	6.3 (5.9; 7.0)	6.7 (6.1; 7.3)	6.6 (6.4; 7.3)
High	6.7 (6.4; 7.2)	7.5 (7.2; 8.0)	7.2 (7.0; 8.0)	7.2 (6.3; 8.2)

eTable 2 Disability-free life expectancy estimates according to wealth in white people, England and United States 2002-2013

	Men		Women	
	England (ELSA) Years (95%CI)	US (HRS) Years (95%CI)	England (ELSA) Years (95%CI)	US (HRS) Years (95%CI)
**Age 50**				
Poorest wealth	22.7 (22.9; 23.3)	22.6 (21.6; 23.5)	24.4 (24.3; 25.3)	24.2 (23.3; 25.1)
Middle wealth	27.6 (27.1; 28.1)	28.0 (27.2; 28.7)	30.0 (29.5; 30.5)	30.1 (29.5; 30.7)
Richest wealth	30.9 (30.3; 31.1)	31.2 (30.9; 31.9)	33.1 (32.8; 33.3)	33.1 (32.6; 33.8)
**Age 60**				
Poorest wealth	14.5 (14.3; 15.0)	14.8 (14.4; 15.2)	16.2 (16.1; 16.9)	15.7 (15.3; 16.1)
Middle wealth	18.9 (18.6; 19.3)	19.5 (19.1; 20.0)	20.8 (20.6; 21.0)	20.9 (20.6; 21.4)
Richest wealth	22.0 (21.5; 22.5)	22.3 (21.8; 22.8)	23.8 (23.6; 22.4)	24.0 (23.5; 24.4)
**Age 70**				
Poorest wealth	8.3 (8.1; 8.6)	8.1 (7.7; 8.7)	9.2 (8.9; 9.7)	9.0 (8.6; 9.4)
Middle wealth	11.4 (11.2; 11.6)	11.7 (11.3; 12.2)	12.8 (12.7; 13.1)	13.0 (12.5; 13.6)
Richest wealth	13.7 (13.6; 13.8)	14.3 (13.9; 14.6)	15.4 (15.1; 15.5)	15.4 (14.8; 16.0)
**Age 80**				
Poorest wealth	4.3 (4.1; 4.6)	4.1 (3.8; 4.5)	4.8 (4.3; 5.1)	4.2 (3.8; 4.4)
Middle wealth	5.8 (5.7; 5.9)	6.2 (5.8; 6.7)	6.9 (6.4; 7.0)	6.8 (6.4; 7.4)
Richest wealth	7.6 (7.4; 7.7)	7.9 (7.4; 8.4)	8.9 (8.4; 9.2)	8.7 (8.3; 9.4)

eTable 3 Disability-free life expectancy estimates according to education in white people, England and United States 2002-2013

	Men		Women	
	**England Years (95%CI)**	**US Years (95%CI)**	**England Years (95%CI)**	**US Years (95%CI)**
**Age 50**				
Low	24.9 (24.4; 25.5)	24.1 (22.7; 25.5)	26.7 (26.4; 27.3)	25.6 (24.8; 26.4)
Middle	28.6 (27.9; 29.2)	28.6 (27.8; 29.3)	30.7 (30.4; 31.2)	29.9 (29.1; 30.7)
High	30.7 (30.1; 32.3)	31.1 (30.4; 31.7)	33.2 (32.5; 34.4)	32.9 (32.2; 33.6)
**Age 60**				
Low	16.3 (15.8; 16.9)	16.6 (15.7; 17.2)	18.2 (17.9; 18.6)	16.7 (15.9; 17.4)
Middle	20 (19.4; 20.4)	19.9 (19.3; 20.5)	21.7 (21.4; 22.2)	20.9 (20.5; 21.4)
High	21.9 (21.2; 23.1)	22.6 (21.7; 23.1)	23.9 (23.2; 25.1)	23.6 (23.0; 24.7)
**Age 70**				
Low	9.6 (9.2; 10.1)	9.2 (8.7; 9.6)	10.6 (10.4; 11.1)	9.3 (8.8; 9.6)
Middle	12.4 (11.4; 13.0)	12.2 (11.9; 12.4)	13.7 (13.4; 14.2)	12.9 (12.6; 13.2)
High	13.9 (13.1; 14.8)	14.2 (13.4; 14.8)	15.5 (14.8; 16.6)	14.9 (14.2; 15.7)
**Age 80**				
Low	4.9 (4.6; 5.2)	5.0 (4.6; 5.5)	5.5 (5.3; 6.0)	4.9 (4.5; 5.5)
Middle	6.9 (6.6; 7.2)	6.7 (6.2; 7.2)	7.8 (7.3; 8.2)	6.8 (6.4; 7.4)
High	7.8 (7.3; 8.2)	8.0 (7.6; 8.9)	8.5 (5.5; 9.7)	8.2 (7.7; 9.4)

eTable 4 Disability-free life expectancy according to the relative index of inequalities for social class, England and United States 2002-2013

	Men		Women	
	**England (ELSA) Years (95%CI)**	**US (HRS) Years (95%CI)**	**England (ELSA) Years (95%CI)**	**US (HRS) Years (95%CI)**
**Age 50**				
Low grade RII=0.85	24.3 (23.9; 25.0)	25.2 (24.3; 25.7)	25.5 (24.9; 26.1)	26.0 (25.4; 26.3)
Middle grade RII=0.5	27.0 (26.5; 27.5)	27.6 (26.7; 27.9)	28.5 (28.0; 28.8)	28.5 (28.0; 28.7)
High grade RII=0.15	29.8 (29.1; 30.2)	29.7 (29.3; 30.6)	31.3 (30.7; 31.9)	31.0 (30.7; 31.4)
**Age 60**				
Low grade RII=0.85	15.9 (15.3; 16.3)	16.9 (16.6; 17.4)	17.1 (16.5; 17.5)	17.2 (16.7; 17.6)
Middle grade RII=0.5	18.2 (17.5; 18.6)	18.9 (18.6; 19.4)	19.6 (19.1; 19.8)	19.5 (19.1; 19.7)
High grade RII=0.15	20.9 (20.2; 21.2)	21.0 (20.7; 21.6)	22.2 (21.7; 22.5)	21.9 (21.5; 22.4)
**Age 70**				
Low grade RII=0.85	9.2 (8.8; 9.6)	10.2 (9.8; 10.4)	9.6 (9.0; 9.9)	10.3 (9.7; 10.5)
Middle grade RII=0.5	11.3 (10.8; 11.7)	11.6 (11.2; 11.8)	12.0 (11.4; 12.3)	12.0 (11.5; 12.3)
High grade RII=0.15	12.8 (11.9; 13.3)	13.3 (13.0; 13.7)	13.7 (13.1; 14.4)	13.8 (13.4; 14.4)
**Age 80**				
Low grade RII=0.85	4.6 (4.2; 4.9)	5.2 (5.0; 5.4)	4.9 (4.6; 5.4)	4.7 (4.2; 5.3)
Middle grade RII=0.5	5.6 (5.1; 6.0)	6.3 (5.8; 6.5)	6.3 (5.8; 6.7)	6.5 (6.2; 6.7)
High grade RII=0.15	6.8 (6.3; 7.2)	7.5 (7.3; 8.0)	7.3 (6.6; 8.0)	7.2 (6.5; 7.5)

eTable 5 Disability-free life expectancy according to the relative index of inequalities for education, England and United States 2002-2013

	Men		Women	
	England (ELSA) Years (95%CI)	US (HRS) Years (95%CI)	England (ELSA) Years (95%CI)	US (HRS) Years (95%CI)
**Age 50**				
Low RII=0.85	23.8	24.1	25.6	24.9
	(23.5; 24.5)	(23.6; 24.7)	(24.9; 26.1)	(24.0; 25.4)
Middle RII=0.5	26.8	27.6	28.9	28.8
	(26.4; 27.4)	(27.2; 28.1)	(28.5; 29.4)	(28.2; 29.2)
High RII=0.15	29.9	30.8	32.1	32.5
	(29.5; 30.9)	(30.2; 31.5)	(31.6; 32.9)	(31.8; 33.1)
**Age 60**				
Low RII=0.85	15.6	15.9	17.4	16.3
	(15.2; 15.9)	(15.5; 16.4)	(16.7; 17.8)	(15.9; 16.8)
Middle RII=0.5	18.3	19.0	20.0	19.9
	(18.1; 18.8)	(18.7; 19.4)	(19.7; 20.7)	(19.4; 20.4)
High RII=0.15	21.1	22.0	22.7	23.3
	(20.6; 22.0)	(21.6; 22.5)	(22.6; 23.9)	(22.5; 23.9)
**Age 70**				
Low RII=0.85	9.1	9.2	10.0	9.4
	(8.7; 9.4)	(8.9; 9.8)	(9.6; 10.3)	(9.1; 9.8)
Middle RII=0.5	11.2	11.9	12.4	12.5
	(10.9; 11.7)	(11.7; 12.2)	(11.9; 12.7)	(12.1; 12.9)
High RII=0.15	13.1	14.3	14.9	15.0
	(12.9; 14.1)	(13.8; 14.6)	(14.2; 15.7)	(14.5; 15.6)
**Age 80**				
Low RII=0.85	4.6	4.9	5.2	4.8
	(4.3; 4.9)	(4.6; 5.5)	(4.9; 5.6)	(4.4; 5.3)
Middle RII=0.5	6.2	6.4	6.9	6.5
	(5.7; 6.7)	(6.2; 7.0)	(6.7; 7.4)	(6.0; 6.9)
High RII=0.15	7.3	8.2	7.8	8.0
	(6.6; 7.9)	(7.7; 8.7)	(7.3; 9.1)	(7.5; 8.7)
